# Functional characterization and stability improvement of a ‘thermophilic-like’ ene-reductase from *Rhodococcus opacus* 1CP

**DOI:** 10.3389/fmicb.2015.01073

**Published:** 2015-10-01

**Authors:** Anika Riedel, Marika Mehnert, Caroline E. Paul, Adrie H. Westphal, Willem J. H. van Berkel, Dirk Tischler

**Affiliations:** ^1^Interdisciplinary Ecological Center, Environmental Microbiology Group, Institute of Biosciences, Technical University Bergakademie FreibergFreiberg, Germany; ^2^Laboratory of Biochemistry, Wageningen UniversityWageningen, Netherlands; ^3^Department of Biotechnology, Delft University of TechnologyDelft, Netherlands

**Keywords:** biocatalysis, flavoprotein, old yellow enzyme, ene-reductase, thermal stability, *Rhodococcus opacus* 1CP

## Abstract

Ene-reductases (ERs) are widely applied for the asymmetric synthesis of relevant industrial chemicals. A novel ER OYE*Ro*2 was found within a set of 14 putative old yellow enzymes (OYEs) obtained by genome mining of the actinobacterium *Rhodococcus opacus* 1CP. Multiple sequence alignment suggested that the enzyme belongs to the group of ‘thermophilic-like’ OYEs. OYE*Ro*2 was produced in *Escherichia coli* and biochemically characterized. The enzyme is strongly NADPH dependent and uses non-covalently bound FMNH_2_ for the reduction of activated α,β-unsaturated alkenes. In the active form OYE*Ro*2 is a dimer. Optimal catalysis occurs at pH 7.3 and 37°C. OYE*Ro*2 showed highest specific activities (45-50 U mg^-1^) on maleimides, which are efficiently converted to the corresponding succinimides. The OYE*Ro*2-mediated reduction of prochiral alkenes afforded the (*R*)-products with excellent optical purity (*ee* > 99%). OYE*Ro*2 is not as thermo-resistant as related OYEs. Introduction of a characteristic intermolecular salt bridge by site-specific mutagenesis raised the half-life of enzyme inactivation at 32°C from 28 to 87 min and improved the tolerance toward organic co-solvents. The suitability of OYE*Ro*2 for application in industrial biocatalysis is discussed.

## Introduction

Old Yellow Enzymes (OYE) or ene-reductases (ER) are flavoproteins catalyzing the asymmetric reduction of activated C = C bonds through a trans-hydrogenation reaction (Stürmer et al., 2007). Creating up to two stereogenic centers, OYEs are versatile biocatalysts for the biotransformation of cyclic and acyclic enones and enals, α,β-unsaturated dicarboxylic acids and esters, maleimides, terpenoids, nitroalkenes, steroids as well as nitrate esters and nitroaromatics (Leuenberger et al., 1976; Takabe et al., 1992; Vaz et al., 1995; Kataoka et al., 2002, 2004; Kurata et al., 2004; Chaparro-Riggers et al., 2007; Hall et al., 2007; Stückler et al., 2007; Nivinskas et al., 2008; Fryszkowska et al., 2009; Adalbjörnsson et al., 2010; Mueller et al., 2010; Opperman et al., 2010; Toogood et al., 2010). Especially with maleimides, relevant products like the anticonvulsant drugs ethosuximide, phensuximide, and methsuximide can be synthesized.

First OYEs were isolated, purified and biochemically characterized from the yeasts *Saccharomyces carlsbergensis* (Warburg and Christian, 1932) and *S. cerevisiae* (Haas, 1938). Similar ‘classical’ OYEs are found in bacteria and plants (Breithaupt et al., 2001; Williams and Bruce, 2002; Toogood et al., 2010) and usually occur in solution as homodimers or heterodimers (Stott et al., 1993). Crystal structures of members of this group are determined for OYE1 (1OYA, 1OYB, and 1OYC; Fox and Karplus, 1994), PETNR (1H50; Barna et al., 2001), and morphinone reductase (1GWJ; Barna et al., 2002).

A second group of bacterial OYEs was designated as ‘YqjM-like’ or ‘thermophilic-like’ OYEs (Toogood et al., 2010). Members of this group, e.g., *Ts*ER (CAP16804, Opperman et al., 2008), TOYE (ABY93685, Adalbjörnsson et al., 2010), *Gk*OYE (BAD76617, Schittmayer et al., 2011), *Geo*ER (BAO37313, Tsuji et al., 2014), and *Chr*-OYE3 (AHV90721, Xu et al., 2014) are stable at temperatures up to 80°C and range in oligomeric state from dimers to dodecamers.

Recently, two novel ‘thermophilic-like’ OYEs were reported (Litthauer et al., 2014). *Rm*ER from *Ralstonia metallidurans* (ABF11721) was isolated as a monomer, while *Dr*ER from *Deinococcus radiodurans* (AAF11740) was monitored as a homodimer. *Dr*ER and *Rm*ER were not studied for their thermal stability but showed activity optima around 30 and 35°C, respectively (Litthauer et al., 2014).

*Rhodococcus* species are known for large genomes and gene redundancy, thus one can expect to identify novel biocatalysts by means of genome mining of these organisms (McLeod et al., 2006). *Rhodococcus opacus* 1CP is such a model organism for gene redundancy and can serve as a source of valuable oxidoreductases (Tischler et al., 2009, 2010; Gröning et al., 2014; Riedel et al., 2015). Herein, we report on the discovery of fourteen OYEs from the actinobacterium *R. opacus* 1CP. Because of its ‘thermophilic-like’ signature, OYE*Ro*2 was selected for heterologous expression and biochemical characterization. Next to its catalytic features we also studied the thermal and co-solvent stability of OYE*Ro*2. Finally, we changed five consecutive amino acid residues in OYE*Ro*2 to generate the more robust variant OYE*Ro*2a.

## Materials and Methods

### Chemicals and Enzymes

Maleimide, *N*-methylmaleimide, *N*-ethylmaleimide, 2-methyl-*N*-phenylmaleimide, 2-cyclohexenone, 1-cyclohexene-1-carboxaldehyde, 1-acetyl-1-cyclohexenone, ketoisophorone, glucose-6-phosphate dehydrogenase and pyridine nucleotide cofactors were purchased from Sigma–Aldrich (Steinheim, Germany) and Carl Roth (Karlsruhe, Germany). 1-Benzyl-1,4-dihydronicotinamide (BNAH) was synthesized as previously described (Paul et al., 2013). Other (bio)-chemicals were obtained from commercial sources and of the purest grade available.

### Bacterial Strains, Plasmids, and Gene Synthesis

Plasmids used in this study are listed in **Table 1**. Both genes, *oyeRo2* and *oyeRo2a* (variant), were synthesized by Eurofins MWG GmbH (Ebersberg, Germany). Gene sequences were codon optimized and cloned into a pUC57 and a pEX-K4 vector system, respectively, both flanked by the restriction sites *Nde*I and *Not*I. In the *oyeRo2a* gene, five codons were changed to create the following amino acid replacements: A92R, Q93R, L94I, E95R, and Y96E. The GC content of both genes was adapted to the codon usage of strain *Acinetobacter* sp. strain ADP1 to gain high yields of soluble protein during expression in *Escherichia coli* or, if necessary, in *Acinetobacter* sp. (Oelschlägel et al., 2015). After ligation of the genes into the vector pET16bP (**Table 1**), the resulting recombinant plasmids pS*Ro*OYE2_P01 and pS*Ro*OYE2a_P01 were transformed into *E. coli* BL21 (DE3).

**Table 1:** Plasmids and primers used in this study.

Plasmids	Relevant characteristic(s)	Source
pET16bP	pET16bP with additional multiple cloning site; allows expression of recombinant proteins with N-terminal His_10_-tag	U. Wehmeyer^∗^
pUC57_OYE*Ro*2	pUC57 vector (2.710-kb) with additional multiple cloning site; Amp^r^ and *oyeRo2* (1.098-kb *Nde*I/*Not*I fragment) as insert	Eurofins MWG operon
pEX-K4_OYE*Ro*2a	pEX-K4 vector (2.507-kb) with additional multiple cloning site; Km^r^ and *oyeRo2a* (1.098-kb *Nde*I/*Not*I fragment) as insert	Eurofins MWG operon
pS*Ro*OYE2_P01	*oyeRo2* of *Rhodococcus opacus* 1CP (1,098-kb *Nde*I/*Not*I fragment) cloned into pET16bP	This study
pS*Ro*OYE2a_P01	*oyeRo2a* of *R. opacus* 1CP (1.098-kb *Nde*I/*Not*I fragment) cloned into pET16bP	This study

*^∗^Personal communication*.

### Heterologous Expression and Protein Purification

Recombinant ERs were obtained as N-terminal His_10_-tagged soluble proteins. OYE*Ro*2 and variant OYE*Ro*2a were expressed from BL21 cells using 3-L-baﬄed flasks with 500 mL of LB medium (100 μg mL^-1^ ampicillin, 50 μg mL^-1^ chloramphenicol) containing 0.5 M NaCl, 0.2% glucose and 1 mM betaine (LBNB; Oganesyan et al., 2007) while shaking constantly at 120 rpm. Cultures were incubated at 30°C until OD_600_ reached 0.5, followed by an 18 h induction with 0.05 mM isopropyl-β-D-thiogalactopyranoside at 25°C. Cells were harvested (5,000 × *g*; 30 min), resuspended in 50 mM phosphate buffer (KH_2_PO_4_/Na_2_HPO_4_; pH 7.1) and disrupted three times by French-press (1,500 psi, < 10°C). Cell debris was removed by centrifugation at 50,000 × *g* for 30 min at 4°C. Protein purification was performed with two tandem 1 mL HisTrap FF columns (GE Healthcare; Tischler et al., 2009). Purified proteins were concentrated using an ultrafiltration device with a MWCO of 5 kDa (Vivaproducts). Protein aliquots were stored at -20°C in 25 mM potassium/sodium phosphate buffer (pH 7.1), containing 40% (vol/vol) glycerol. Purity and subunit molecular mass of recombinant proteins was estimated by sodium dodecyl sulfate polyacrylamide gel electrophoresis (SDS-PAGE).

### Analytical Gel Filtration

Hydrodynamic properties of OYE*Ro*2 and OYE*Ro*2a were analyzed at room temperature by usage of an Äkta Explorer FPLC system (Pharmacia Biotech) applying a Superdex 200 HR 10/30 column (GE Healthcare). As a mobile phase 50 mM phosphate buffer (pH 7.1) containing 150 mM NaCl was used. To assess the influence of redox state of the protein on its mobility, the elution buffer was supplied with 200 μM NADPH. The flow rate was 0.5 mL min^-1^ and per run 100 μL of protein was applied. The following proteins were used as markers: cytochrome C (12.3 kDa), ribonuclease A (14.4 kDa), myoglobin (17.8 kDa), α-chymotrypsinogen (25 kDa), carbonic anhydrase (27 kDa), ovalbumin (43 kDa), ovalbumin-dimer (86 kDa), BSA (68 kDa), BSA-dimer (136 kDa), conalbumin (79 kDa), lipoamide dehydrogenase (102 kDa), aldolase (148 kDa), phenol-2-hydroxylase (152 kDa), catalase (232 kDa), ferritin (467 kDa), and vanillyl-alcohol oxidase (510 kDa). Dextran blue (2,000 kDa) was used to determine the void volume (*V*_0_) and acetone (58 Da, 1% in water) was used to determine the total accessible volume (*V*_t_). Apparent *M*_r_ values of ERs were obtained from a graph where the partition coefficient (*K*_av_) of the standard proteins was plotted against log *M*_r_ (**Figure 3B**, inset).

### Flavin Analysis

Flavin content of ERs was determined spectrophotometrically from absorption scans (300 to 600 nm). The molar absorption coefficient for free FMN was applied (ε_445_ = 12.5 mM^-1^cm^-1^; Whitby, 1953). The identity of non-covalently bound flavin (Tao et al., 2008) was determined by the procedure adapted earlier (Tischler et al., 2010). Flavins were analyzed by RP-HPLC, equipped with a Eurospher C18 column (125-mm length, 4 mm diameter, 5 μm particle size, 100 Å pore size; Knauer, Germany). Elution was performed with 50 mM sodium acetate (pH 5.0) and a linear gradient of 15–60% methanol lasting 20 min (flow 0.7 mL min^-1^). As standards, FAD (net retention volume *V*_R_ = 8.9 mL), FMN (*V*_R_ = 11.7 mL) and riboflavin (*V*_R_ = 14.5 mL) were used.

### Enzyme Activity

Activity of ERs was determined spectrophotometrically by following the consumption of NADPH, NADH, or BNAH at 340 nm (ε_340_ = 6.22 mM^-1^cm^-1^). 1 Unit (U) of OYE*Ro*2 activity is defined as the amount of enzyme that catalyzes the conversion of 1 μmol NAD(P)H per minute. The standard assay (1.0 mL) was performed at 25°C in 50 mM phosphate buffer (pH 7.1) containing 140 μM NADPH and 1 mM maleimide or 1 mM *N*-ethylmaleimide, respectively. The reaction was started through the addition of enzyme to a final concentration of 30 nM. Background activity with oxygen was measured in the absence of maleimide and was subtracted from the total activity yield.

The substrate specificity of OYE*Ro*2 was analyzed by replacing maleimide in the standard assay with the following compounds (1 mM): 2-cyclohexen-1-one, 3-methyl-2-cyclohexenone, 2-methyl-2-cyclohexenone, 1-cyclohexene-1-carboxylic acid, 1-cyclohexene-1-carboxaldehyde, 1-acetyl-1-cyclohexenone, ketoisophorone (KIP), *N*-methylmaleimide, *N*-ethylmaleimide, and 2-methyl-*N*-phenylmaleimide. All substrates were applied from 100 mM stock solutions in ethanol. In case of KIP the assay was performed at 365 nm using a molar absorption coefficient of 3.51 mM^-1^cm^-1^ (Fu et al., 2012).

Optimum temperature for OYE*Ro*2 activity was determined by pre-incubation of the standard assay mixture (without enzyme) at temperatures between 10 and 50°C for 10 min and initiating the reaction by the addition of enzyme. The optimum pH for OYE*Ro*2 activity was determined by usage of phosphate buffer in the standard assay mixture with pH values varying between 5.0 and 9.0, and the activity measured at 25°C.

Thermal stability was measured by incubation of 30 nM (low concentration) or 7 μM (high concentration) enzyme in 50 mM phosphate buffer (pH 7.1) at temperatures between 1 and 50°C. Samples were taken periodically and assayed for residual OYE activity using the standard assay. The pseudo first-order rate constants for enzyme inactivation (*k*_in_) obtained from the double-exponential equation fits were converted to half-lifes using the equation: half-life = ln(2/*k*_in_).

Determination of co-solvent activity was performed under standard assay conditions by the addition of 10% (vol/vol) organic solvents [ethanol, methanol, dimethyl sulfoxide (DMSO), dimethylformamide (DMF), acetonitrile, acetone]. To determine the dependency of enzyme activity on co-solvent concentration, the amounts of co-solvent in the assay was varied for ethanol (0-30%) and for DMSO (0-40%). To measure the time-dependent enzyme stability in presence of co-solvent, the enzyme was preincubated in 20% ethanol (in 25 mm KH_2_PO_4_/Na_2_HPO_4;_ pH 7.1) at 25°C for certain times prior to the assay.

### Biotransformation of Enones and Maleimides

Conversion of cyclic enones was performed using 1-mL sealed glass vials containing the following components in a final volume of 500 μL: 25 mM potassium/sodium phosphate buffer (pH 7.1), 1 mM cyclic enone, 200 μM NADP^+^, 5 mM glucose-6-phosphate, 0.02 μM glucose-6-phosphate dehydrogenase (110 U mg^-1^) and 1 μM enzyme. Reactions were performed for 24 h at 20°C in vials constantly shaken at 550 rpm. Remainder of substrates and products formed were extracted for 10 min with ethylacetate (1:1) and analyzed via gas chromatography equipped with a flame ionization detector (United Technology Packard, model 437A, Downers Grove, ILL, USA). As a stationary phase a 10% Chromosorb W column (1 m × 2 mm) was used. Nitrogen was applied as the mobile phase (25 mL min^-1^). The system was operated in isothermal mode. Temperatures of injector and detector were set to 250°C. The column temperature was adjusted for optimal separation to 95°C for 2-cyclohexen-1-one, to 110°C for 1-cyclohexene-1-carboxaldehyde, 2-methyl-2-cyclohexenone and 1-acetyl-1-cyclohexenone, to 115°C for 3-methyl-2-cyclohexenone and to 140°C for KIP.

Conversion of maleimides was performed as described above lacking the regeneration system. Reactions were performed for merely 15 min at 20°C. Determination of remainder of maleimides and formed products was performed via RP-HPLC. As stationary phase a C18 Eurosphere II-column (250 mm × 4.6 mm) and as mobile phase a mixture of methanol and water (40:60), acidified with 20 mM H_3_PO_4_, was applied.

### Stereochemistry

Enzymatic reactions with prochiral substrates were performed by incubation of 3.4 μM OYE*Ro*2 in 50 mM KPi, pH 7.0, containing 1 mM substrate, 1.25 mM NADPH and 0.5% DMSO for 2 h 30 min at 30°C. After extraction with ethyl acetate and evaporation, the solid components were dissolved in an *n*-heptane/isopropanol mixture (95:5) and the solution obtained was used for injection. For GC analysis, after identical incubations, the ethyl acetate layer was dried with MgSO_4_, centrifuged (13000 rpm, 1 min) and liquid layer was transferred to GC vials.

HPLC analyses were carried out on a Shimadzu 20-series HPLC. The enantiomeric excess of 2-methyl-*N*-phenylmaleimide was determined on a Chiralcel OD column (250 mm × 4.6 mm) running in *n*-heptane/isopropanol (95:5) at 40°C at a flow rate of 1 mL/min.

GC analyses were carried out on a Shimadzu GC-2010 gas chromatograph equipped with an FID. (**A)** A Chiral Dex CB column (25 m × 0.32 mm × 0.25 μm) was used for the separation of KIP. The injection temperature was 250°C, helium was used as a carrier gas; linear velocity: 25 cm/s, split ratio: 30. (**B)** A Chiral Dex CB column (60 m × 0.25 mm × 0.25 μm) was used for the separation of 2-methyl-*N*-phenylmaleimide. The injection temperature was 250°C, helium was used as a carrier gas; linear velocity: 34 cm/s, split ratio: 30. (**C)** A Meta-DEX β column (25 m × 0.25 mm × 0.25 μm) was used for the separation of 2-methylcyclohexenone. The injection temperature was 240°C, helium was used as a carrier gas; linear velocity: 24.8 cm/s, split ratio: 50. GC column oven temperature programs are listed in the Supporting Information.

### Phylogenetic Analysis

The genome of the actinobacterium *R. opacus* 1CP was investigated for OYE family members through similarity searches applying BLASTp (Altschul et al., 1990, 1997). Pairwise sequence analysis was performed with EMBOSS Needle (Rice et al., 2000). The maximum likelihood distance tree was computed by Mega 6.06-mac applying Clustal W for multiple sequence alignment (500 bootstrap replications). The gene sequences of *oyeRo2* and *oyeRo2a* have been deposited at GenBank under the accession numbers KR349311 and KR349312.

### Structural Modeling

An alignment was made of the sequences of OYE*Ro*2 and OYE*Ro*2a from *R. opacus* 1CP and of *Ts*ER from *Thermus scotoductus* (50% amino acid sequence identity). Using this alignment and the available dimeric structure of *Ts*ER (PDB-id: 3hf3) as template, dimeric structural models including FMN were obtained for OYE*Ro*2 and OYE*Ro*2a using the MODELLER program version 9.15 (Sali and Blundell, 1993). One hundred comparative models were generated, after which the models with lowest corresponding DOPE scores (Eswar et al., 2006) were selected. Tetrameric structure models were obtained by applying symmetry operators provided in the pdb file of *Ts*ER (Supplementary Figure S4). Pymol (PyMOL Molecular Graphics System, Version 1.5.0.4) was used to generate images.

## Results

### Functional Annotation of Ene-reductases from *Rhodococcus opacus* 1CP and Creation of Variant OYE*Ro*2a

**FIGURE 1:**
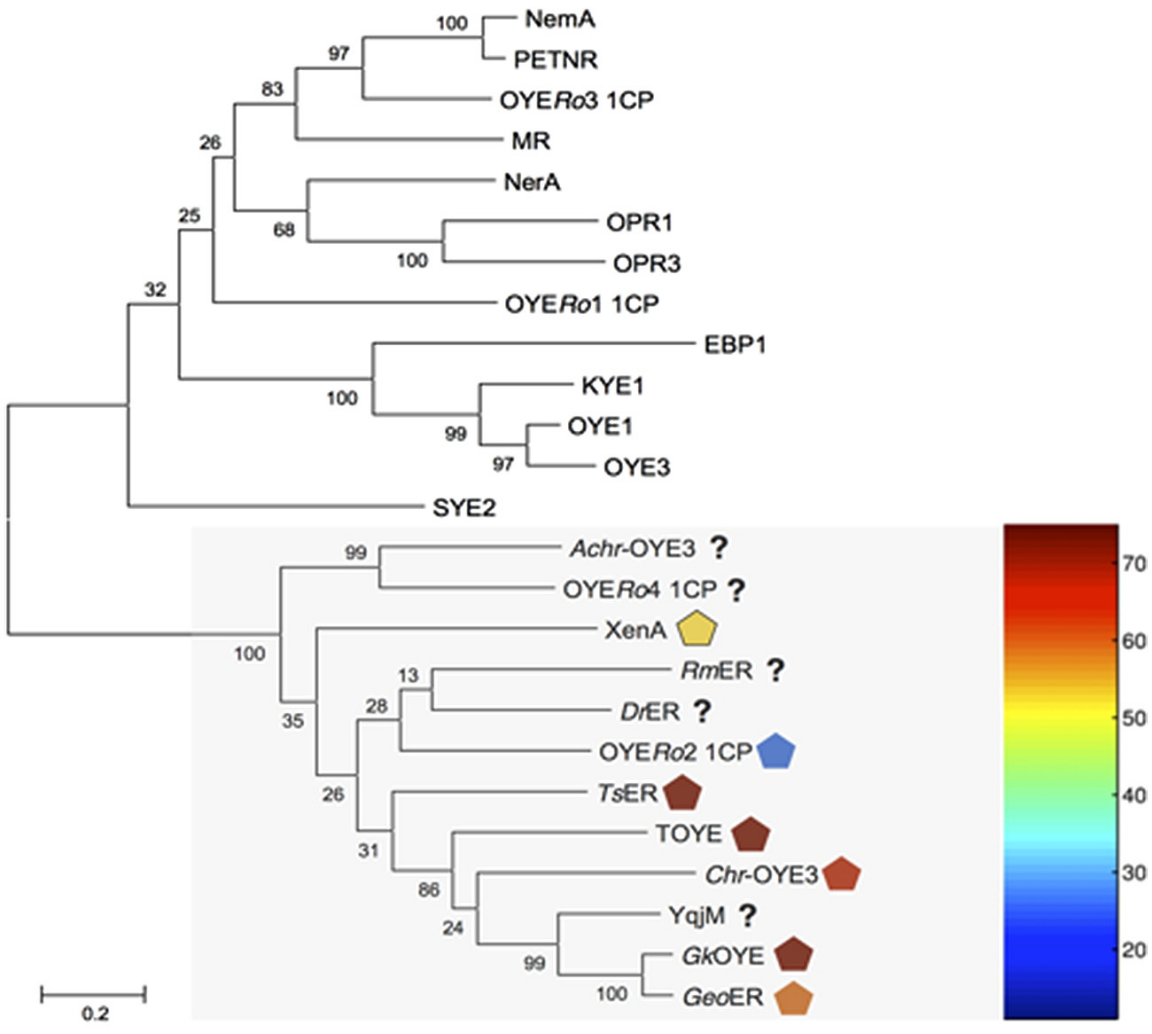
**Dendrogram of amino acid sequences of old yellow enzymes (OYEs) from *Rhodococcus opacus* 1CP and characterized OYE enzymes.** The maximum likelihood distance tree was solved by usage of Mega 6.06-mac with Clustal W alignment method. Test of phylogeny was performed with 500 bootstrap replications, and resulting numbers are indicated. ‘Thermophilic-like’ OYEs are indicated by a light gray box. Melting temperatures of ‘thermophilic-like’ OYEs are indicated by symbols colored with respect to the thermogram. NCBI accession numbers are in parentheses. OYE1: *Saccharomyces pastorianus* (*carlsbergensis*, CAA37666), OYE3: *S. cerevisiae* (CAA97878), KYE1: *Kluyveromyces lactis* (AAA98815), EBP1: *Candida albicans* (AAA18013), OPR1: *Solanum lycopersicum* (CAB43506), OPR3: *S. lycopersicum* (CAC21424), NerA: *Agrobacterium tumefaciens* (CAA74280), MR: *Pseudomonas putida* (AAC43569), OYE*Ro*1 and OYE*Ro*3: *R. opacus* 1CP, NemA: *Escherichia coli* (BAA13186), PETNR: *Enterobacter cloacae* (AAB38638), SYE2: *Shewanella oneidensis* (AAN55487), *Achr*-OYE3: *Achromobacter* sp. JA81 (AFK73187), OYE*Ro*2 and OYE*Ro*4: *R. opacus* 1CP, *Rm*ER: *Ralstonia (Cupriavidus) metallidurans* CH34 (ABF11721), *Dr*ER: *Deinococcus radiodurans* R1 (AAF11740), *Ts*ER: *Thermus scotoductus* (CAP16804), XenA: *P. putida* (AAF02538), TOYE: *Thermoanaerobacter pseudethanolicus* (ABY93685), *Chr*-OYE3: *Chryseobacterium* sp. CA49 (AHV90721), YqjM: *Bacillus subtilis* (BAA12619), *Gk*OYE: *Geobacillus kaustophilus* (BAD76617) and *Geo*ER: *Geobacillus* sp. #30 (BAO37313).

Genome mining of *R. opacus* 1CP identified fourteen open reading frames (ORFs) encoding for putative OYE. Multiple sequence alignment showed that four of these proteins (OYE*Ro*1-4) shared regions conserved in ‘classical’ or in ‘thermophilic-like’ OYEs (Toogood et al., 2010). Sequence comparison with previously characterized ERs established that OYE*Ro*1 and OYE*Ro*3 belong to the ‘classical’ subclass while OYE*Ro*2 and OYE*Ro*4 group within the ‘thermophilic-like’ subclass (**Figure 1**). A dendrogram including the other ten OYE*Ro*-enzymes (Supplementary Figure S1) revealed that these ERs cluster in additional subclasses, which are not structurally described yet. OYE*Ro*2 drew our special attention since typically rhodococci do not encode for thermophilic proteins and that provided the motivation to investigate this enzyme.

**FIGURE 2:**
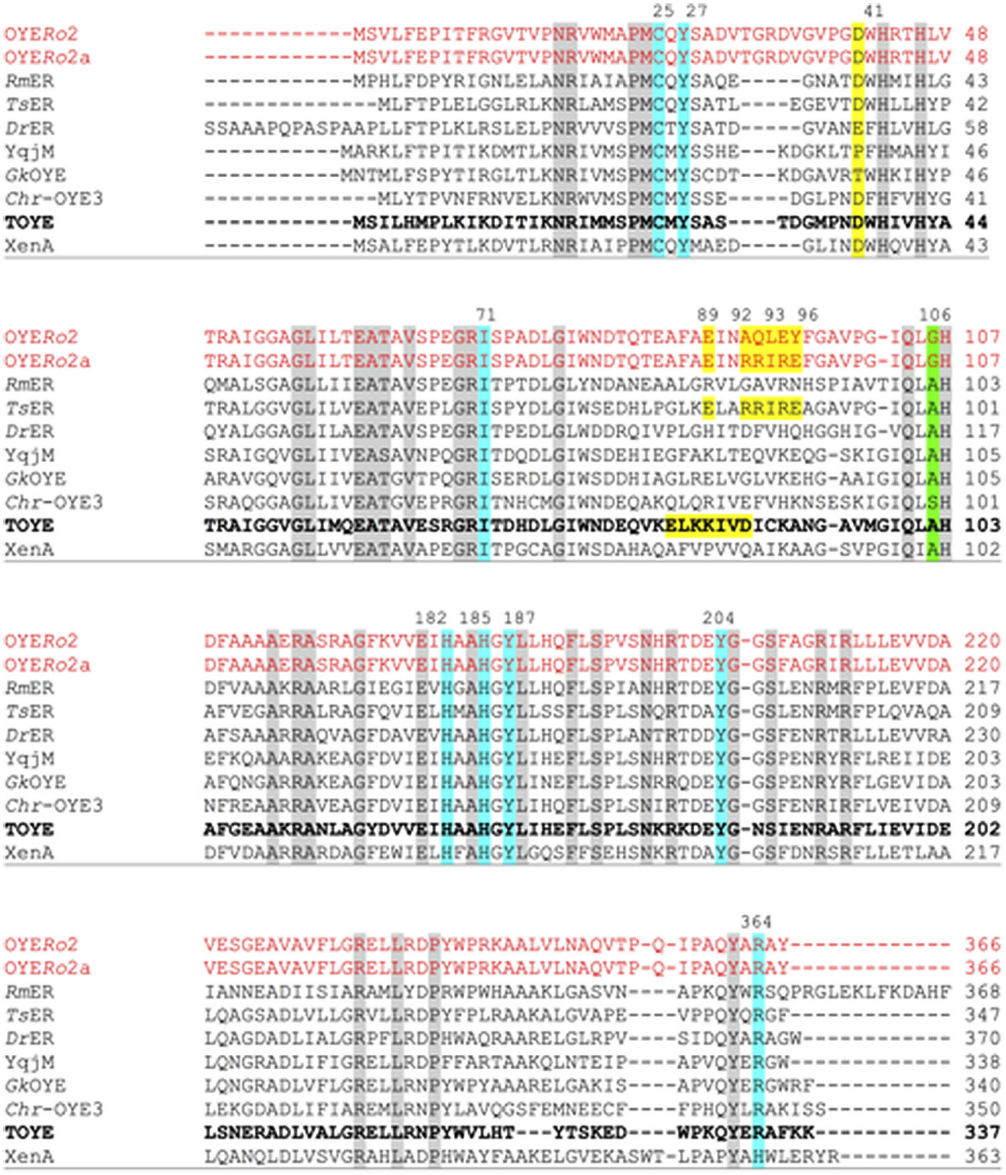
**Multiple sequence alignment of OYE*Ro*2 and OYE*Ro*2a with eight ‘thermophilic-like’ OYE homologs.** Highly conserved residues are shaded in gray, conserved residues involved in the active site are shaded in blue and green. The salt bridges of *Ts*ER and TOYE are shaded in yellow. Amino acids that were replaced by site-directed mutagenesis of OYE*Ro*2 are also shaded in yellow in the wildtype and in the protein variant OYE*Ro*2a. Amino acid numbering of conserved residues is given according to the TOYE sequence (bold font). Other amino acid numberings are given according to each protein sequence. NCBI accession numbers and organism sources: *Rm*ER: *R. (Cupriavidus) metallidurans* CH34 (ABF11721), *Dr*ER: *D. radiodurans* R1 (AAF11740), *Ts*ER: *T. scotoductus* (CAP16804), XenA: *P. putida* (AAF02538), TOYE: *T. pseudethanolicus* (ABY93685), *Chr*-OYE3: *Chryseobacterium* sp. CA49 (AHV90721), YqjM: *B. subtilis* (BAA12619) and *Gk*OYE: *G. kaustophilus* (BAD76617).

Pairwise sequence alignment of OYE*Ro*2 revealed highest similarity to the thermostable ERs *Ts*ER (50% identity; 62% similarity), TOYE (45%; 59%), *Gk*OYE (48%; 61%), and *Geo*ER (47%; 59%). A close relationship was also highlighted with the mesophilic ERs *Dr*ER (49%; 61%) and *Rm*ER (46%; 59%) (Litthauer et al., 2014). The sequence comparisons also established that the OYE*Ro*2 active site residues Cys25, Tyr27, Ile71, His182, His185, Tyr187, Tyr204, and Arg364 are conserved (**Figure 2**). Interestingly, OYE*Ro*2 contains a glycine at position 106 (**Figure 2**, green shadow). This residue is mostly an alanine in ‘thermophilic-like’ ERs and strictly conserved as a tryptophan in ‘classical’ OYEs. Switching the bulky tryptophan to alanine or glycine could be the reason for an enlarged active site volume of ‘thermophilic-like’ OYE (Adalbjörnsson et al., 2010; Toogood et al., 2010).

‘Thermophilic-like’ OYEs show great differences in thermal stability (**Figure 1**). Higher melting temperatures were observed for *Ts*ER and TOYE compared to XenA and YqjM (Opperman et al., 2010; Yanto et al., 2010). XenA and YqjM are stabilized through plain salt bridges containing single pairs of charged amino acids (Opperman et al., 2010). On the contrary, *Ts*ER was found to contain a complex salt bridge network at the dimerization interface (Opperman et al., 2010). Sequence analysis suggested that a similar network is present in TOYE (**Figure 2**). In *Ts*ER the complex five-residue salt bridge: Asp37^#^ (^#^from adjacent monomer), Glu85, Arg88, Arg89, and Glu92 is responsible for the linkage between helices α1^#^ and α2 (**Figure 2**; Opperman et al., 2010). From homology modeling of OYE*Ro*2 using the structure of *Ts*ER as template, it was noticed that OYE*Ro*2 most likely lacks this salt bridge. Hence, the variant OYE*Ro*2a was created with the substitutions A92R, Q93R, L94I, E95R, and Y96E. In summary, a novel ER OYE*Ro*2 was identified in *R. opacus* 1CP that shares highest sequence identity with the thermostable ER *Ts*ER. Furthermore, a characteristic five-residue salt bridge was introduced for creation of the variant OYE*Ro*2a.

### Cloning, Expression, Purification and Hydrodynamic States of OYE*Ro*2 and OYE*Ro*2a

Both *oyeRo2* and *oyeRo2a* (mutant) genes, each with a size of 1101 bp, were adapted to the codon usage of strain *Acinetobacter* sp. strain ADP1 (allowing for higher gene expression levels within *E*. *coli*). The restriction sites *Nde*I and *Not*I were introduced N-terminal and C-terminal in both genes, which were successfully cloned into the His_10_-tag containing pET16bP vector. OYE*Ro*2 and OYE*Ro*2a were obtained in soluble form by expression of the genes in *E. coli* BL21 (DE3) as host using high salt LBNB-media (0.5 M NaCl). From *oyeRo2* gene expression, a cell dry weight of 1.87 g and 9.0 mg soluble OYE*Ro*2 protein per liter culture was obtained. This is about 7% of the total soluble protein. Similar results were obtained for OYE*Ro*2a.

**FIGURE 3:**
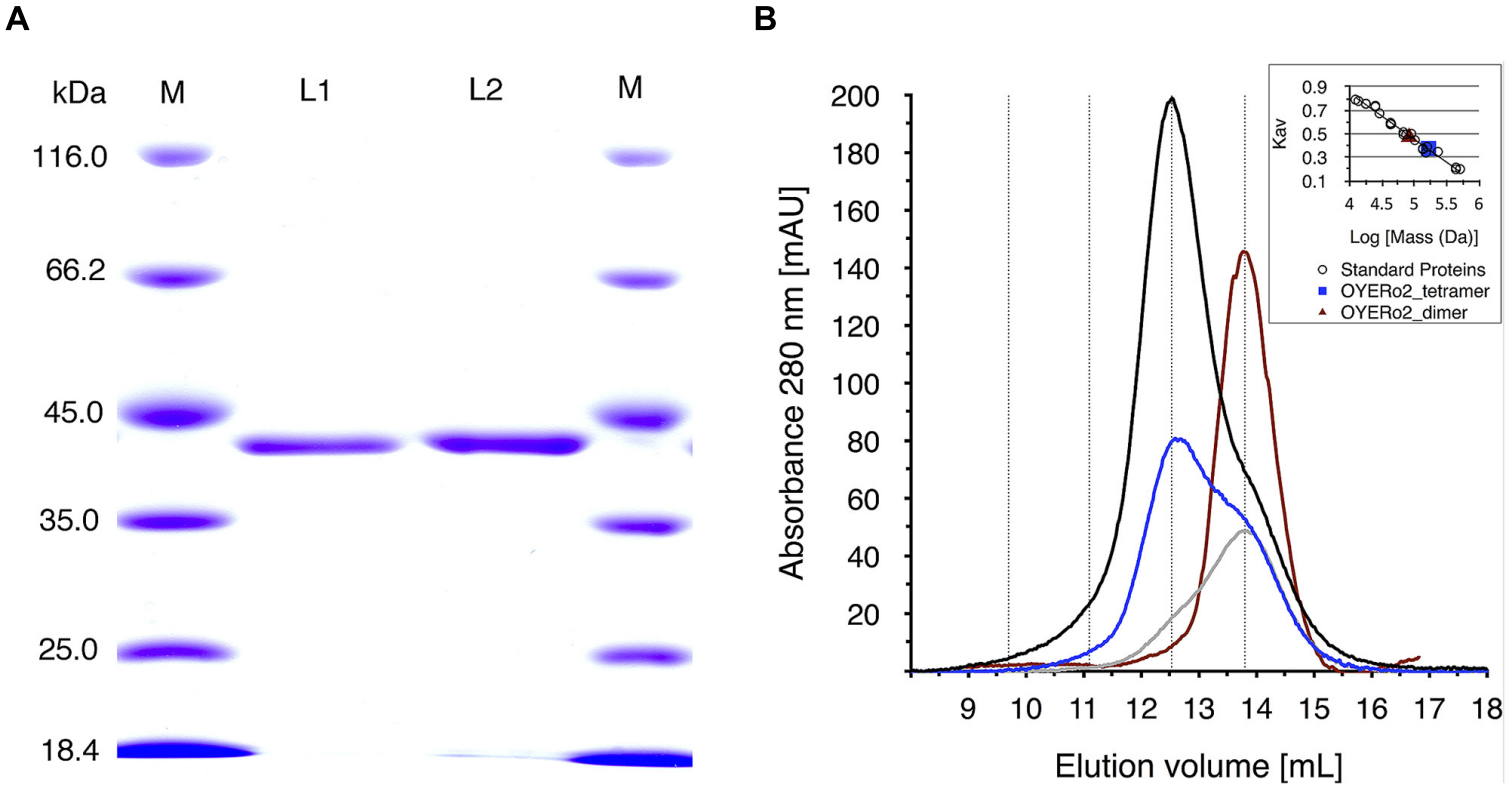
**(A)** SDS-PAGE analysis of samples of OYE*Ro*2 and OYE*Ro*2a. Lane 1: marker proteins, lane 2: purified OYE*Ro*2, lane 3: purified OYE*Ro*2a, lane 4: marker proteins. **(B)** Analytical gel filtration of purified OYE*Ro*2 samples eluted from a Superdex 200 HR 10/30 column. Black line: 119 μM (high concentration), blue line: 59 μM (medium concentration), gray line: 30 μM (low concentration), red line: 30 μM enzyme in presence of 200 μM NADPH. The inset shows the calibration curve obtained from elution of standard proteins. Due to the calibration the following retention volumes are stressed by dotted lines: dimer: 13.8 mL, tetramer: 12.5 ml, octamer: 11.1 mL, hexadecamer: 9.7 mL. ND: no significant activity/conversion detected under steady-state conditions.

His_10_-tagged proteins were purified via immobilized nickel ion chromatography. SDS-PAGE analysis indicated an apparent subunit molecular mass of 42 kDa (**Figure 3A**), as expected from the deduced amino acid sequences (387 amino acid residues, with theoretical molecular masses of 41,615 Da). Both OYE*Ro*2 and OYE*Ro*2a had a bright yellow color, indicative for a tightly bound flavin (see Spectral Analysis). Purified proteins were found to be stable in 25 mM phosphate buffer (pH 7.1) and were stored in this buffer containing 40% glycerol at -20°C.

Analytical gel filtration revealed that both proteins occur as oligomers in solution. At high protein concentration, the enzymes mainly exist as tetramers, whereas at lower protein concentration, the equilibrium of species shifts toward the dimeric state (**Figure 3B**). The oligomeric state of OYE*Ro*2 turned out to depend also on the interaction with NADPH and/or flavin redox state. In the presence of NADPH, the enzyme fully dissociated to a homodimer (**Figure 3B**).

### Spectral Analysis

**FIGURE 4:**
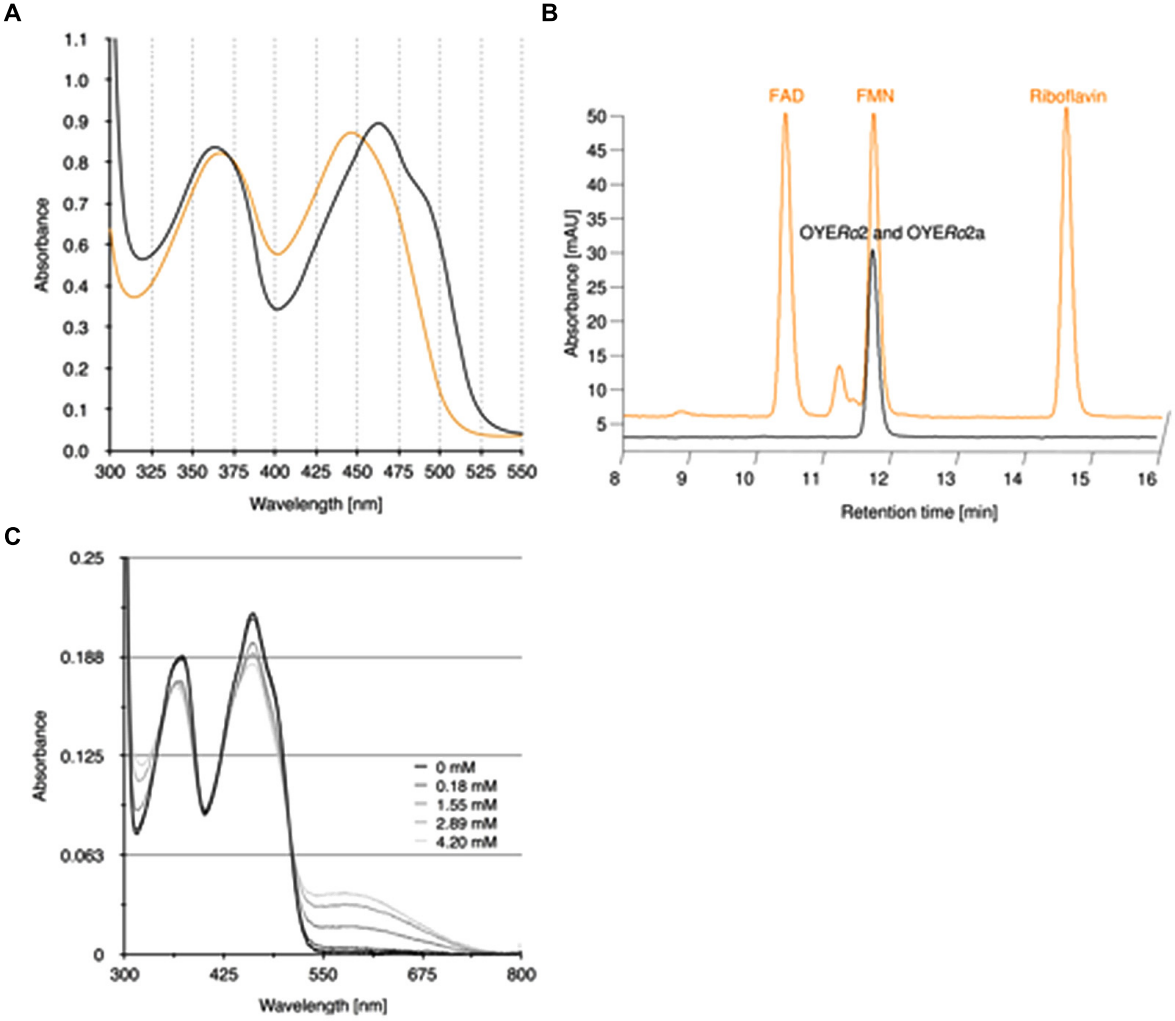
**Binding of flavin cofactor to OYE*Ro*2 and OYE*Ro*2a. (A)** UV-VIS absorption spectra of 100 μM purified OYE*Ro*2 and of 100 μM purified OYE*Ro*2a (orange lines, overlapping) and a similar amount of free FMN (black line). **(B)** RP-HPLC on flavin samples extracted from purified OYE*Ro*2 and OYE*Ro*2a (black lines, overlapping) and on flavin standards FAD, FMN and riboflavin (orange line, with offset). **(C)** Spectral changes in OYE*Ro*2 upon titration with *p*-chlorophenol (0**–**4.2 mM) at 15°C. The enzyme concentration was 13.8 μM in 50 mM phosphate buffer (pH 7.1).

The purified OYE*Ro*2 proteins revealed flavin absorption maxima at 362 and 462 nm (**Figure 4A**). Denaturation of proteins with trichloroacetic acid and subsequent spectral analysis of the yellow supernatant (**Figure 4A**) allowed a calculation of the amount of flavin bound to the protein. A protein-flavin ratio of 1:1 was obtained, which implies that 1 mol flavin binds non-covalently to 1 mol of OYE protein. From the absorbance differences between protein-bound flavin and free flavin, molar absorption coefficients, ε_462_ = 10.9 mM^-1^cm^-1^ and ε_462_ = 11.4 mM^-1^cm^-1^, for protein-bound flavin could be estimated for OYE*Ro*2 and OYE*Ro*2a, respectively. The type of flavin cofactor was identified as FMN using RP-HPLC (**Figure 4B**).

OYE*Ro*2 forms charge-transfer complexes with phenolic ligands. Next to 4-hydroxybenzaldehyde, which is a well-known inhibitor of ‘classical’ OYEs (Abramovitz and Massey, 1976; Fox and Karplus, 1994), the enzyme showed affinity for several halophenols. As an example, the flavin spectral perturbations induced through binding of *p*-chlorophenol are presented in **Figure 4C**. The appearance of the long-wavelength absorption around 600 nm suggests that OYE*Ro*2 binds this inhibitor in its phenolate form (Abramovitz and Massey, 1976).

### Kinetic Characterization

**FIGURE 5:**
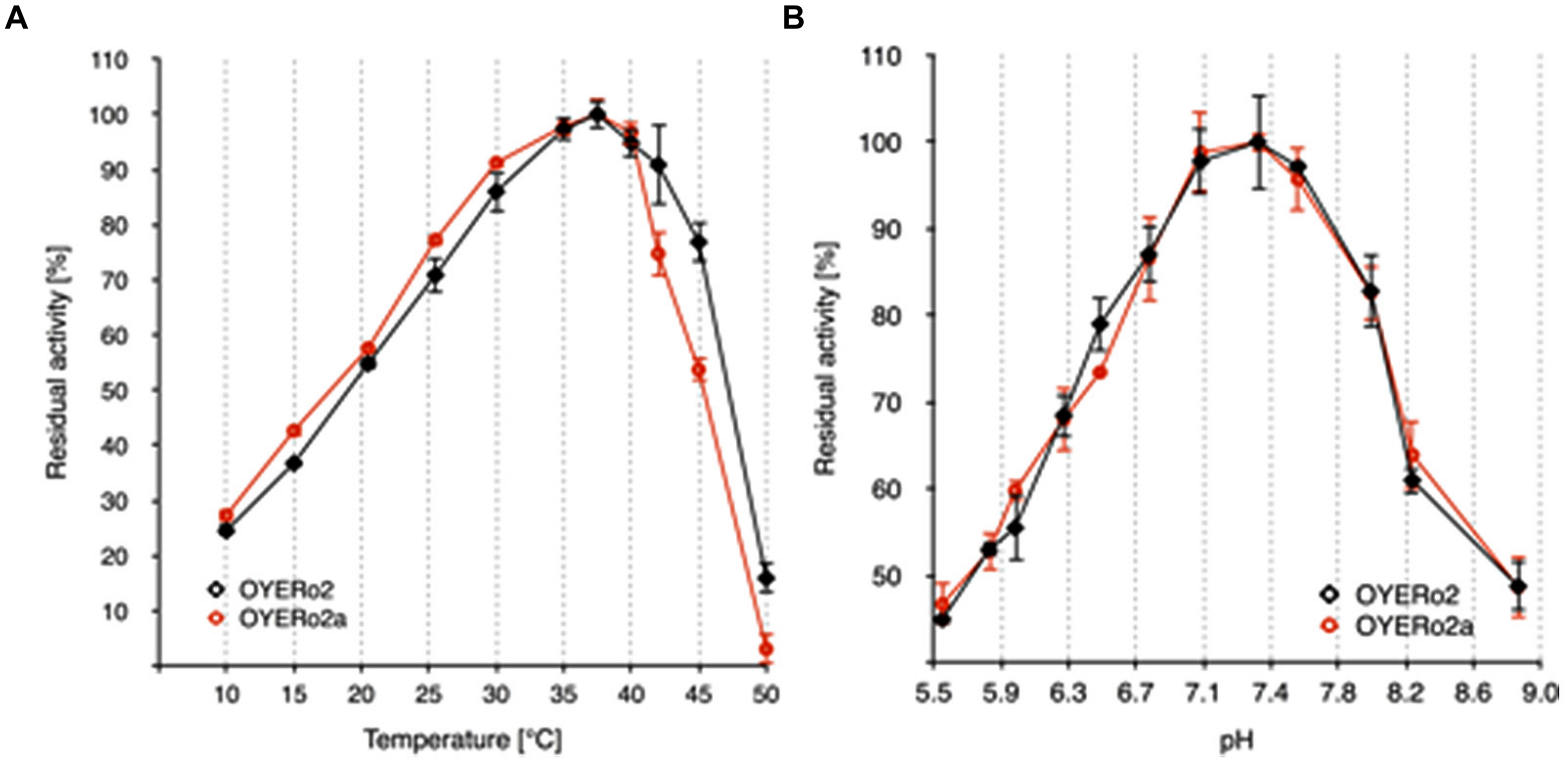
**Temperature and pH optima of purified OYE*Ro*2 (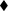) and purified OYE*Ro*2a (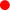). (A)** Normalized activity-temperature plot: 140 μM NADPH and 1 mM maleimide in 50 mM phosphate buffer (pH 7.1) were incubated at various temperatures (10-50°C) for 10 min. The reaction was started through the addition of 30 nM enzyme (not tempered). **(B)** Normalized activity-pH plot: 140 μM NADPH, 1 mM maleimide in 50 mM phosphate buffers (pH 5.5 to 8.8) and 30 nM enzyme, measured at 25°C.

The OYE*Ro*2 proteins showed highest activities at temperatures between 35 and 40°C (**Figure 5A**) and pH values between 7.0 and 7.6 (**Figure 5B**).

OYE*Ro*2 had a strong preference for NADPH over NADH as electron donor (**Table 2**). The enzyme was also active with the synthetic nicotinamide cofactor BNAH (Paul et al., 2013). Steady-state kinetic analysis with saturating concentrations of *N*-ethylmaleimide established that the *V*_max_ value for BNAH is in the same range as found for NADPH and that the Michaelis constant (*K*_m_) is more comparable to that of NADH. As a result, the catalytic efficiency of OYE*Ro*2 with BNAH is intermediate regarding to NADPH and NADH (**Table 2**).

**Table 2:** Kinetic parameters of OYE*Ro*2 proteins toward nicotinamide cofactors and maleimides.

Proteins	Substrates	*V*_max_ (U mg^-1^)	*K*_m_ (μM)	*k*_cat_ (s^-1^)	*k*_cat_/*K*_m_ (μM^-1^ s^-1^)
OYE*Ro*2	NADPH^a^	49.6	15.4	34.8	2.3
	NADH^a^	1.5	354	1.0	0.003
	BNAH^b^	45.2	380	31.7	0.08
	Maleimide^c^	46.4	3.0	32.5	10.8
	*N*-ethylmaleimide ^c^	50.2	3.1	35.2	11.4
OYE*Ro*2a	NADPH^a^	51.5	16.7	36.2	2.2
	Maleimide^c^	49.6	4.4	34.9	7.9

*Standard assay at 25°C with 25 mM phosphate buffer (pH 7.1), OYERo2 and OYERo2a (12.6 μg ml^-1^) and ^a^1 mM maleimide or ^b^1 mM N-ethylmaleimide or ^c^140 μM NADPH. All values have a maximal experimental error of 10%.*

OYE*Ro*2 and OYE*Ro*2a showed similar activities and affinities with NADPH and maleimide. Hence, the catalytic efficiencies of both enzymes are matchable.

### Substrate Specificity

OYE*Ro*2 showed strong and almost identical activity with maleimide substrates (**Table 3**). The specific activities with maleimides (45–51 U mg^-1^ at 25°C) count to the highest reported for ERs among LeOPR with *N*-ethylmaleimide (41.3 U mg^-1^; Straßner et al., 1999), TOYE with *N*-methylmaleimide (40.3 U mg^-1^; Adalbjörnsson et al., 2010), *Chr*-OYE3 with 2-methyl-*N*-phenylmaleimide (52.9 U mg^-1^, Xu et al., 2014), and Bac-OYE2 with 2-methylmaleimide (44 U mg^-1^; Zhang et al., 2014). OYE*Ro*2 was much less active with cyclic enones. Here, 2-cyclohexen-1-one (**5**) showed the highest conversion rate. OYE*Ro*2 was also poorly active with 1-cyclohexen-1-carboxaldehyde (**11**; **Table 3**).

**Table 3:**
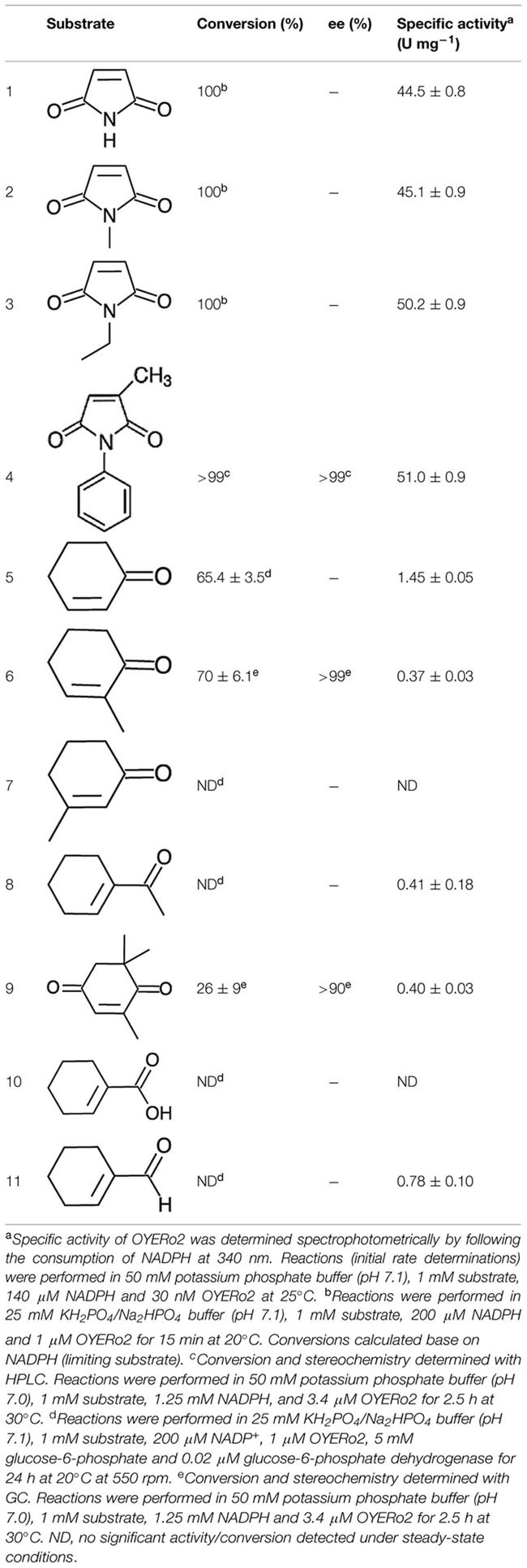
Activity of OYE*Ro*2 with α,β-unsaturated alkenes.

Substrate conversion was studied by incubating the enzyme with maleimides for 15 min and cyclic enones for 24 h at 20°C (see Experimental). Maleimides (**1, 2, 3, 4**) were completely converted to the corresponding succinimides (**Table 3**). Partial conversion was noticed for 2-cyclohexen-1-one (**5**), 2-methyl-2-cyclohexen-1-one (**6**) and KIP (**9**) to give the corresponding cycloketones (**Table 3**). No detectable conversion was monitored for 3-methyl-2-cyclohexen-1-one (**7**) and for 1-cyclohexen-1-carboxylic acid (**10**), respectively. This behavior was also observed for *Ts*ER, TOYE, *Dr*ER, and *Rm*ER. Under the conditions applied, OYE*Ro*2 showed some NADH oxidation but hardly any conversion with 1-acetyl-cyclohexenone (**8**) and 1-cyclohexene-1-carboxaldehyde (**11**), similarly as described for the reaction of an ER from *T. thermophilus* with 1,4-benzoquinone (Steinkellner et al., 2014). NADPH oxidation was observed and calculated from spectrophotometric analysis under aerobic conditions when no substrate was applied to the assay.

### Stereochemistry

OYE*Ro*2 showed excellent enantioselectivity in the reactions with 2-methyl-*N*-phenylmaleimide (**4**), and 2-methyl-2-cyclohexen-1-one (**6**). With both prochiral substrates an enantiomeric excess (*ee*) of more than 99% was observed for the (*R*)-product (**Table 3** and Supporting Information). Identical results were obtained with the OYE*Ro*2a variant, or when NADPH was replaced by the artificial cofactor BNAH. The somewhat lower enantiopurity of (6*R*)-levodione (**Table 3**), the product of the enzymatic reduction of KIP (**9**), is due to a non-enzymatic racemisation process progressing during the incubation time (Fryszkowska et al., 2009).

### Thermal and Co-solvent Stability

**FIGURE 6:**
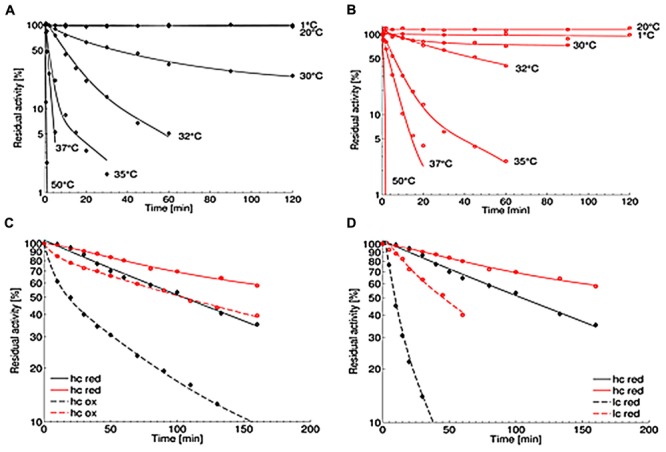
**Thermal stability of purified OYE*Ro*2 (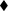) and OYE*Ro*2a (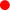). (A,B)** Thermal stability was measured applying the standard assay. Protein was incubated in 50 mM phosphate buffer (pH 7.1) at various temperatures (1–50°C) and added to the assay in a final concentration of 30 nM. **(C)** Inactivation at 32°C with constant high protein concentrations of 7 μM (hc) and varying NADPH concentrations: 1 mM (red; continuous lines) and 0 mM (ox; dashed lines). **(D)** Inactivation at 32°C with various protein concentrations under reducing conditions: 7 μM protein and 1 mM NADPH (hc; continuous lines) and 30 nM protein and 0.14 mM NADPH (lc, dashed lines).

The thermal stability of the OYE*Ro*2 enzymes was analyzed at pH 7.1 at temperatures between 1 and 50°C. While the wildtype protein OYE*Ro*2 was only stable up to 20°C and lost more than 90% activity after 60 min at 32°C (**Figure 6A**), the variant protein OYE*Ro*2a was rather stable at 30°C and still remained 40% activity after 60 min at 32°C (**Figure 6B**). The thermal inactivation at 32°C is presented as a function of flavin redox state (**Figure 6C**) and of protein concentration (**Figure 6D**). At 32°C and 30 nM protein concentration, the half-lifes of enzyme inactivation of OYE*Ro*2 and OYE*Ro*2a were 28 and 87 min, respectively. The thermal stability of OYE*Ro*2 and OYE*Ro*2a strongly increased at higher protein concentration (**Figure 6D**), and an even higher stability was observed when the enzymes were incubated in the presence of NADPH (**Figure 6C**). In summary, under all conditions applied, the engineered variant was considerably more thermo-resistant than the wildtype enzyme.

**FIGURE 7:**
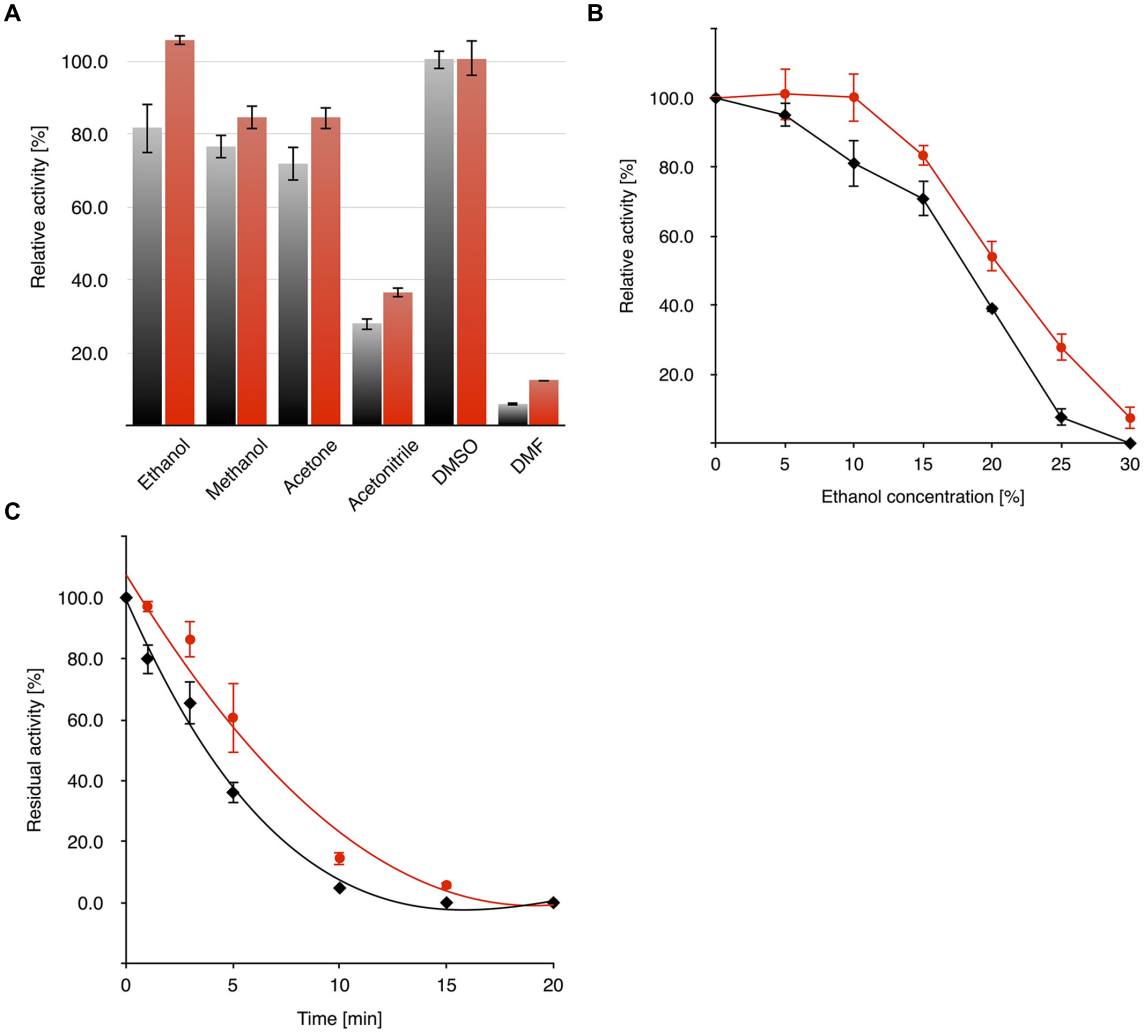
**Activity and stability of OYE*Ro*2 (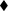) and OYE*Ro*2a (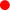) in the presence of organic co-solvents. (A)** Activity of enzymes against 10% (vol/vol) of selected organic co-solvents was measured under assay conditions applying 140 μM NADPH, 25 mM phosphate buffer (pH 7.1) and 30 nM enzyme without additional incubation time. **(B)** Dependency of enzyme activity on ethanol concentration (0–30%). **(C)** Residual activity after pre-incubation (0–20 min) of the enzyme with 20% ethanol.

The activity of OYE*Ro*2 and OYE*Ro*2a in different organic co-solvents was determined from standard assays containing 10% (v/v) co-solvent. It was observed that both enzymes display less activity with acetonitrile (<40% activity) and DMF (<15% activity) than with other co-solvents (80–100% activity; **Figure 7A**). The activity of the OYE*Ro*2 enzymes was also addressed as a function of co-solvent concentration. OYE*Ro*2 retained less activity in ethanol (**Figure 7B**) and DMSO (Supplementary Figure S3) compared to OYE*Ro*2a. Furthermore, upon storage for 20 min in 20% ethanol, OYE*Ro*2 lost its activity more rapidly than OYE*Ro*2a (**Figure 7C**). To summarize, in all co-solvents the variant protein performed better than the wild-type enzyme.

## Discussion

Four from fourteen putative ERs from *R. opacus* 1CP were found to cluster in the two known and structurally described subclasses of OYE: ‘classical’ and ‘thermophilic-like.’ The remaining ten OYEs from strain 1CP clustered together with many more putative OYEs (biochemically and structurally not described yet) in additional subclasses. These findings may hint toward the existence of more OYE subclasses or other enzyme classes, which share high sequence identities with OYE. This assumption can be followed up by means of biochemical characterization and structural investigations of all OYE*Ro-*proteins. In this study we have chosen the ‘thermophilic-like’ OYE*Ro*2 for closer characterization since it was a promising candidate for biocatalysis due to its putative stability and substrate spectrum.

### Quaternary Structure of OYE*Ro*2

The association-dissociation behavior of OYE*Ro*2 (**Figure 3B**) might clarify the range of quaternary structures reported for other ‘thermophilic-like’ OYEs (Kitzing et al., 2005; Adalbjörnsson et al., 2010; Spiegelhauer et al., 2010; Schittmayer et al., 2011; Tsuji et al., 2014; Xu et al., 2014). For monomeric *Rm*ER and dimeric *Dr*ER, a possible influence of the used His-tag on the association behavior of the proteins was not excluded (Litthauer et al., 2014). Here, we could demonstrate that an N-terminal His_10_-tag does not prevent oligomerization of OYE*Ro*2. Furthermore, we could show that flavin reduction by NADPH also influences the oligomerization state of OYE*Ro*2. This finding suggests that nicotinamide cofactor binding and/or flavin reduction introduces conformational perturbations that are transmitted to the protein surface. This is supported by the observation that the thermal stability of OYE*Ro*2 also depends on the protein concentration and the flavin redox state (**Figures 6C,D**).

### Biocatalysis

OYE*Ro*2 has a strong cofactor preference for NADPH. Nevertheless, the usage of inexpensive BNAH might be attractive for future applications of OYE*Ro*2 since the turnover number with this artificial cofactor is rather high (**Table 2**).

OYE*Ro*2 was very active with small and bulky maleimides (**Table 3**). This promiscuity and the fact that the enzyme is highly enantioselective (**Table 3**) makes OYE*Ro*2 an excellent candidate for the production of valuable (asymmetric) succinimides. Anticonvulsant drugs like ethosuximide, phensuximide, and methsuximide can be produced by means of such a biocatalyst. Furthermore, succinimides are core structural units in biologically active compounds as hirsutellones with antibacterial activity, haterumaimides with antitumor activity and tandospirones with anxiolytic and antidepressant effects (Chauhan et al., 2013; Vízcaíno-Milla et al., 2015). Sustainable production of these compounds will be of high interest, since enantiorich succinimides can easily be transformed into pyrrolidines, γ-lactams, and γ-lactones (Kokotos, 2013; Vízcaíno-Milla et al., 2015). For *Ts*ER, the specific activity with maleimides was not reported, but excellent conversions were shown (Paul et al., 2013). Further, high conversions were observed with a range of synthetic nicotinamide cofactors. Thus, additional kinetic studies of *Ts*ER with maleimides would be helpful to discriminate both enzymes.

OYE*Ro*2 seems less suitable for the biotransformation of acyclic enones (**Table 3**). The specific activity of *Ts*ER with 2-cyclohexen-1-one is reported to be 28 U mg^-1^ (Opperman et al., 2010), which is about nineteen times higher than that of OYE*Ro*2 (1.45 U mg^-1^). Although this difference is partly caused by the variation in assay temperature (65 and 20°C, respectively), it is evident that OYE*Ro*2 is more suited for the conversion of maleimides.

### OYE*Ro*2a – a More Robust Enzyme Variant

**FIGURE 8:**
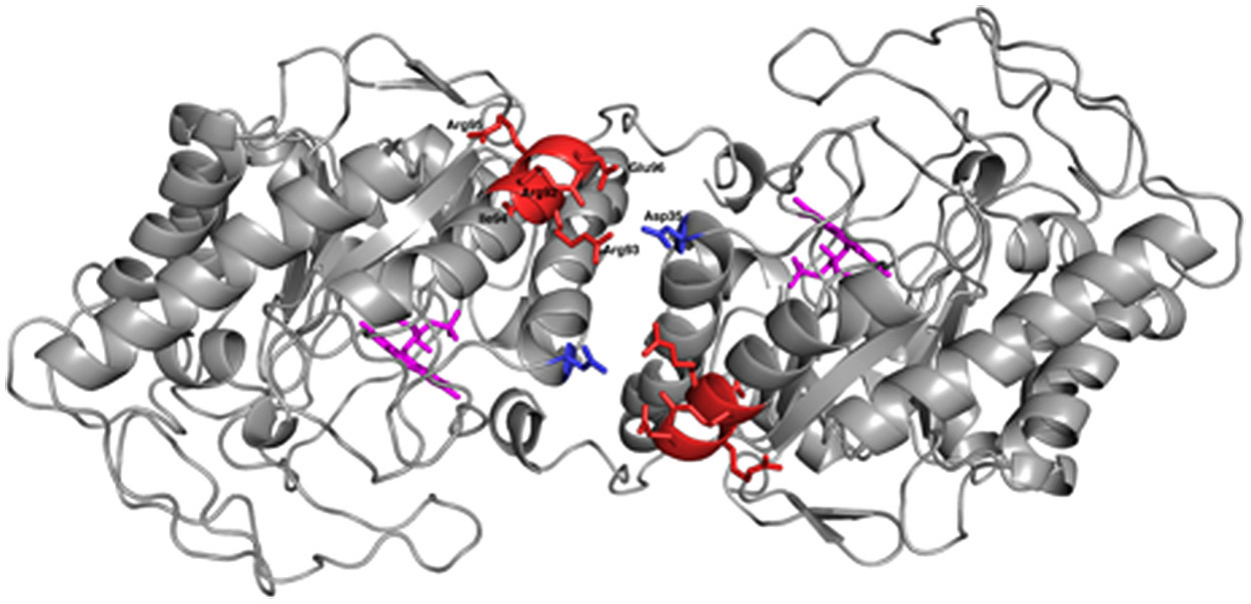
**Cartoon diagram of the dimeric model structure of *R. opacus* 1CP variant OYE*Ro*2a (PyMOL Molecular Graphics System, Version 1.5.0.4).** OYE*Ro*2a is represented in its oxidized form with the FMN cofactor (purple), showing the five residues from the introduced characteristic salt bridge (red) and the Asp35 residue from the adjacent monomer (blue).

OYE*Ro*2a (**Figure 8**) is a protein variant with similar catalytic properties as the wildtype enzyme. It displays a higher thermal stability and better tolerance toward organic solvents (**Figures 6** and **7**). This stability improvement can be explained through an increase in inter-subunit interaction at the dimerization interface (**Figure 8**), which was predicted from homology modeling of OYE*Ro*2 and *Ts*ER. Since *Ts*ER contains two more salt bridges at the dimerization interface, it is conceivable that engineering these salt bridges into OYE*Ro*2a will result in an even more robust OYE*Ro*2 biocatalyst that has excellent turnover rates with maleimides.

## Conclusion

In this paper we have described the properties of a novel ER OYE*Ro*2, which was discovered by genome mining of the actinobacterium *R. opacus* 1CP. OYE*Ro*2 is highly enantioselective in the conversion of prochiral alkenes and reduces a wide range of maleimides to the corresponding succinimides with activities up to 50 U mg^-1^ (**Table 3**). Phylogenetic analysis revealed that OYE*Ro*2 groups within the ‘thermophilic-like’ OYE subclass (**Figure 1**) and shows highest sequence identity (50%) to the thermostable reductase *Ts*ER (*T*_opt_ = 65°C, Opperman et al., 2008). OYE*Ro*2 appeared to be optimally active at 37°C (**Figure 5**) and less thermostable than *Ts*ER (**Figure 6**). These findings prompted us to engineer protein variant OYE*Ro*2a for which we demonstrated more robust properties in all aspects.

## Author Contributions

AR and MM carried out the molecular genetic studies and recombinant protein production. AR, MM, and AW carried out the analytical gel filtration, spectral analysis and kinetics (data acquisition and analysis). Substrate specificity and product analysis was established and carried out by MM and AW. CEP synthesized BNAH and analyzed the stereochemistry. AR, DT, and AW carried out the protein modeling. AR, AW, DT, and WB drafted the manuscript, which was critically revised by all authors. All authors read and approved the final manuscript.

## Conflict of Interest Statement

The authors declare that the research was conducted in the absence of any commercial or financial relationships that could be construed as a potential conflict of interest.
