# Supplementary material for: Functional characterization and stability improvement of a ‘thermophilic-like’ ene-reductase from Rhodococcus opacus 1CP
Source: Front Microbiol. 2015 Oct 1;6:1073. doi: 10.3389/fmicb.2015.01073 (PMC4589676; doi:10.3389/fmicb.2015.01073)
Supplement: Supplementary file 1 [file Data_Sheet_1.PDF]

*Supplementary Material*

# Functional characterization and stability improvement of a 'thermophilic-like' *ene*-reductase from *Rhodococcus opacus* 1CP

**Anika Riedel[1,2*], Marika Mehnert[1], Caroline E. Paul[3], Adrie H. Westphal[2], Willem J.H. van Berkel[2], Dirk Tischler[1,2*]**

[1]Interdisciplinary Ecological Center, Institute of Biosciences, Environmental Microbiology Group, Technical University Bergakademie Freiberg, 09599 Freiberg, Germany

[2]Laboratory of Biochemistry, Wageningen University, 6700ET Wageningen, The Netherlands

[3]Department of Biotechnology, Delft University of Technology, 2628BL Delft, The Netherlands

*** Correspondence:** Anika Riedel and Dirk Tischler, Interdisciplinary Ecological Center, Institute of Biosciences, Environmental Microbiology Group, Technical University Bergakademie Freiberg, 09599 Freiberg, Germany. riedelanika@gmail.com and dirk-tischler@email.de

## 1. Supplementary Figures

In the following figures we highlight the phylogeny of OYE-like amino acid sequences derived of the genome of strain 1CP (Fig. S1), kinetic data mentioned but not displayed in the manuscript (Fig. S2 and S3), as well as a three-dimensional model of the structure of tetrameric mutant OYE*Ro*2a.

From the phylogeny it becomes clear that most of the 14 OYE-like amino acid sequences are putative ene-reductases for which we cannot predict a function yet. Further studies are needed to understand the abundance of these OYE-like sequences in the *R. opacus* 1CP genome and their metabolic role in the microorganism.
The kinetic data displayed in Figures S2 and S3 show that the wild type and mutant enzyme have a similar catalytic behavior. However, as outlined in the manuscript, the mutant has a higher stability against, for example, solvents.
The 3D-structure of OYE*Ro*2a obtained from homology modelling (Fig. S4) is presented to visualize the FMN-protein interaction as well as to display the possible interaction between individual dimers forming the proposed tetramer.

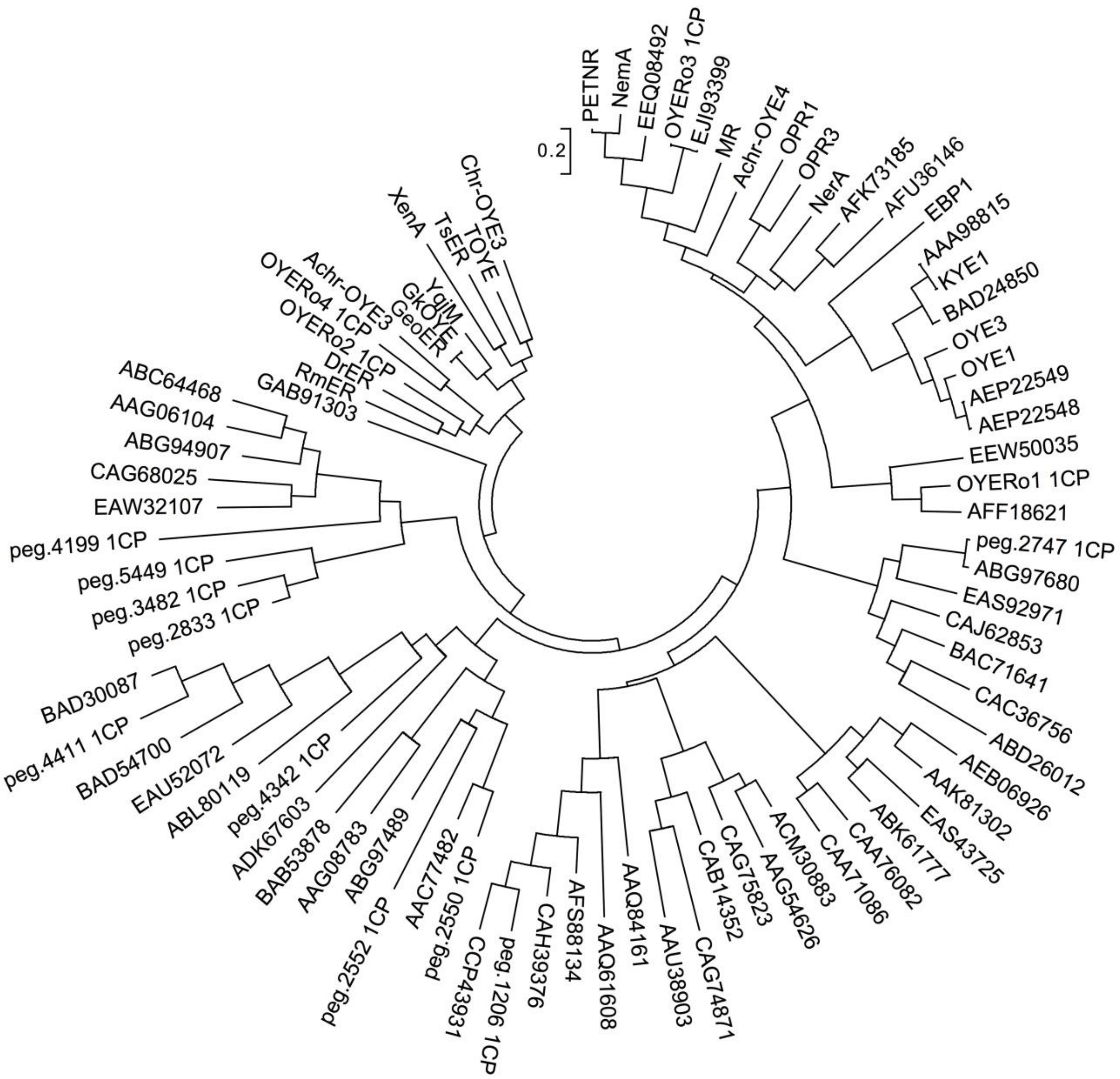


Fig. S1. Dendrogram of amino acid sequences of OYEs from *Rhodococcus opacus* 1CP, characterized OYE enzymes and putative OYEs. The maximum likelihood distance tree was solved by usage of Mega 6.06-mac with Clustal W alignment method. ‚Classical' OYEs from *Rhodococcus opacus* 1CP are OYE*Ro*1 and OYE*Ro*3. ‚Thermophilic-like' OYEs from *Rhodococcus opacus* 1CP are OYE*Ro*2 and OYE*Ro*4. Putative OYEs from *Rhodococcus opacus* 1CP are: peg.2747, peg.1206, peg.2550, peg.2552, peg.4342, peg.4411, peg.2833, peg.3482, peg.5449 and peg.4199. NCBI accession numbers of characterized ‚classical' OYEs are in parentheses: OYE1: *Saccharomyces pastorianus* (*carlsbergensis*, CAA37666), OYE3: *Saccharomyces cerevisiae* (CAA97878), KYE1: *Kluyveromyces lactis* (AAA98815), EBP1: *Candida albicans* (AAA18013), OPR1: *Solanum lycopersicum* (CAB43506), OPR3: *Solanum lycopersicum* (CAC21424), NerA: *Agrobacterium tumefaciens* (CAA74280), MR: *Pseudomonas putida* (AAC43569), NemA: *Escherichia coli* (BAA13186), PETNR: *Enterobacter cloacae* (AAB38638), *Achr*-OYE4: *Achromobacter* sp. JA81 (AFK73188). NCBI accession numbers of putative ‚classical' OYEs are in parentheses: *Yersinia bercovieri*

ATCC43970 (EEQ08492), *Rhodococcus* sp. JVH1 (EJI93399), *Achromobacter* sp. JA81 (AFK73185), *Acinetobacter baumanii* TYTH-1 (AFU36146), *Kluyveromyces lactis* (AAA98815), *Kluyveromyces marxianus* (BAD24850), *Saccharomyces cerevisiae* (AEP22549), *Naumovozyma castellii* (AEP22548), *Corynebacterium efficiens* YS-314 (EEW50035), *Arthrobacter* sp. JBH1 (AFF18621). NCBI accession numbers of characterized ‚thermophilic-like' OYEs are in parentheses: *Rm*ER: *Ralstonia (Cupriavidus) metallidurans* CH34 (ABF11721), *Dr*ER: *Deinococcus radiodurans* R1 (AAF11740), *Achr*-OYE3: *Achromobacter* sp. JA81 (AFK73187), *Ts*ER: *Thermus scotoductus* (CAP16804), XenA: *Pseudomonas putida* (AAF02538), TOYE: *Thermoanaerobacter pseudethanolicus* (ABY93685), *Chr*-OYE3: *Chryseobacterium* sp. CA49 (AHV90721), YqjM: *Bacillus subtilis* (BAA12619), *Gk*OYE: *Geobacillus kaustophilus* (BAD76617) and *Geo*ER: *Geobacillus* sp. #30 (BAO37313). NCBI accession numbers of other putative OYEs are in parentheses: *Rhodococcus jostii* RHA1 (ABG97680), *Mycobacterium* sp. JLS (EAS92971), *Frankia alui* ACN14a (CAJ62853), *Streptomyces avermitilis* MA4680=NBRC14893 (BAC71641), *Streptomyces coelicolor* A3(2) (CAC36756), *Novosphingobium aromaticivorans* DSM12444 (ABD26012), *Coriobacterium glomerans* PW2 (AEB06926), *Closteridium acetobutylicum* ATCC824 (AAK81302), *Photobacterium profundum* 3TLK (EAS43725), *Clostridium novyi* NT (ABK61777), *Moorella thermacetica* (CAA76082), *Clostriudium tyrobutyricum* (CAA71086), *Agrobacterium radiobacter* K84 (ACM30883), *Eschericia coli* 0157:H7 str. (AAG54626), *Pectobacterium atrosepticum* SCRI1043 (CAG74871), *Mannheimia succiniciproducens* MBEL55E (AAU38903), *Streptomyces* sp. HK803 (AAQ84161), *Eschericia coli* 0104:H4 str. 2009EL-2071 (AFS88134), *Burkholderia pseudomallei* K96243 (CAH39376), *Mycobacterium tuberculosis* H37Rv (CCP43931), *Rhodococcus erythropolis* (AAC77482), *Rhodococcus jostii* RHA1 (ABG97489), *Pseudomonas aeruginosa* PAO1 (AAG08783), *Mesorhizobium loti* MAFF303099 (BAB53878), *Olsenella uli* DSM 7084 (ADK67603), *Nocardioides* sp. JS614 (ABL80119), *Rhodobacterales bacterium* HTCC2255 (EAU52072), *Rhizobium* sp. 4-9 (BAD54700), *Pimelobacter simplex* (BAD30087), *Marine Gamma Proteobacterium* HTCC2143 (EAW32107), *Acinetobacter* sp. ADP1 (CAG68025), *Rhodococcus jostii* RHA1 (ABG94907), *Pseudomonas aeruginosa* PAO1 (AAG06104), *Erythrobacter litoralis* HTCC 2594 (ABC64468), *Gordonia rhizosphera* (GAB91303).

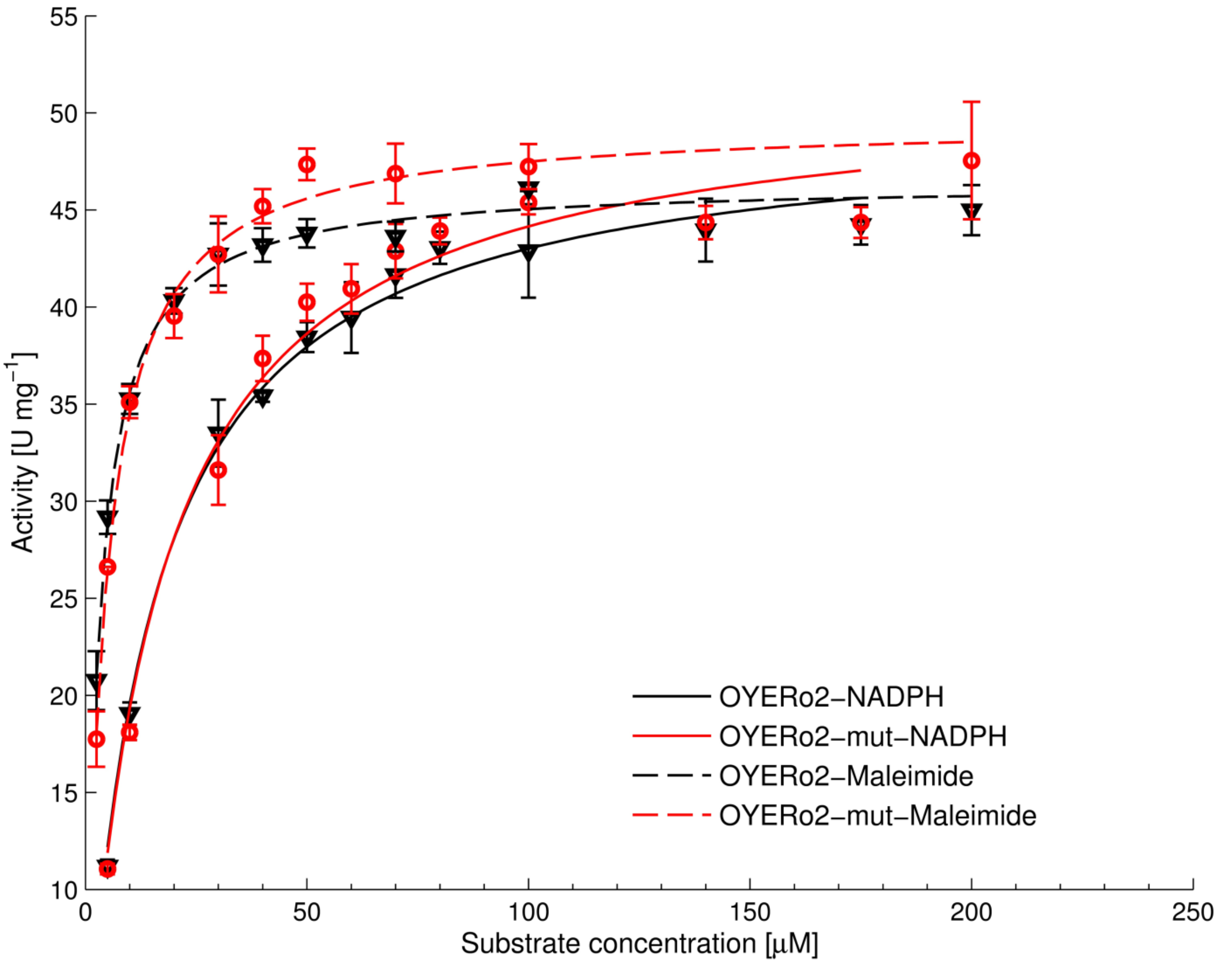


Fig. S2. Michaelis-Menten kinetics of OYE*Ro*2 (▼) and OYE*Ro*2a (○) with NADPH and maleimide as substrates. Kinetic parameters ($K_m$ and $V_{max}$) were determined from the standard assay at 25 °C applying 25 mM phosphate buffer (pH 7.1), OYE*Ro*2 / OYE*Ro*2a (12.6 µg ml$^{-1}$) and 1 mM maleimide with NADPH (0 – 200 µM) (continuous lines) or 140 µM NADPH with maleimide (0 – 100 µM) (dashed lines), respectively. Kinetic parameters were obtained from triplicate measurements by non-linear least-square data fitting using MATLAB version 8.1.0.

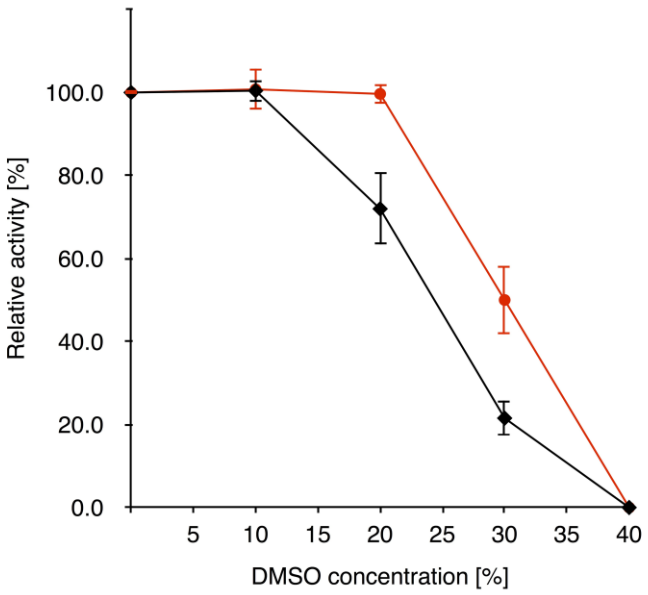


Fig. S3. Dependency of enzyme activity of OYE*Ro*2 (◆) and OYE*Ro*2a (○) on DMSO concentration (0 – 40%). Activity was measured under standard assay conditions applying 140 μM NADPH, 25 mM phosphate buffer (pH 7.1) and 30 nM enzyme without additional incubation time.

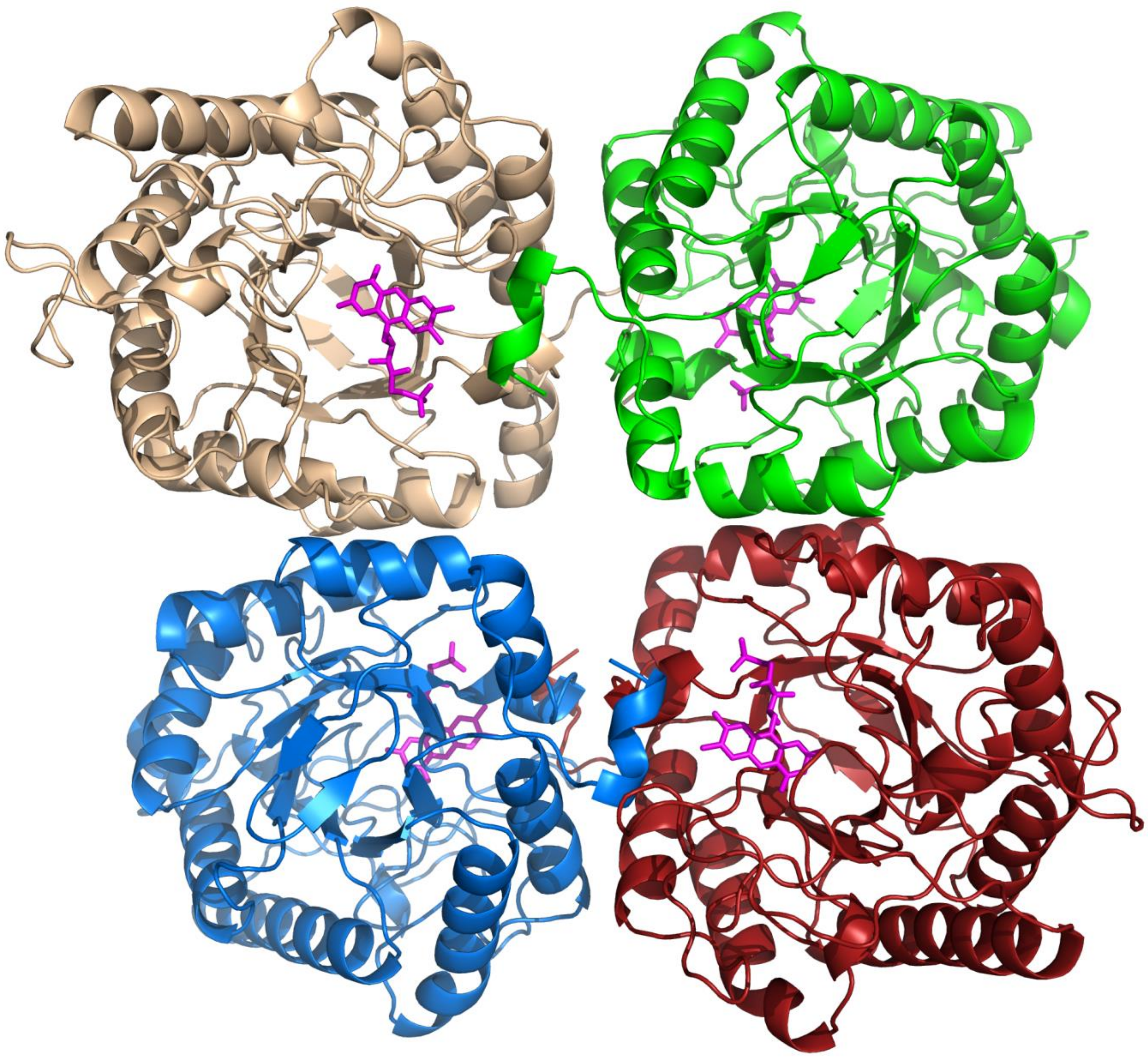

Fig. S4. Cartoon diagram of the tetrameric model structure of *R. opacus* 1CP variant OYE*Ro*2a (PyMOL Molecular Graphics System, Version 1.5.0.4). OYE*Ro*2a is represented in its oxidized form with the FMN cofactor depicted in purple. The four subunits are shown in blue, red, green and beige.

## 2. Supplementary Tables

In the following tables we give detailed information for stereochemistry analysis of the substrates ketoisophorone, 2-methyl-*N*-phenylmaleimide, and 2-methylcyclohexenone. The oven temperature programs of the applied columns A, B and C are shown in Table S1. The obtained retention times of the alkenes and their hydrogenated products are listed in Table S2. And the detailed conversions (%) and enantiomeric excesses of both, OYE*Ro*2 and variant OYE*Ro*2a are listed in Table S3.

Table S1. GC column oven temperature programs

| Column A | Ramp (°C/min) | Temperature (°C) | Time (min) |
|---|---|---|---|
| | - | 110 | 4 |
| | 5 | 130 | |
| | 20 | 220 | 1 |

| Column B | Ramp (°C/min) | Temperature (°C) | Time (min) |
|---|---|---|---|
| | - | 190 | 5 |
| | 25 | 220 | 8 |
| | 25 | 240 | 1 |

| Column C | Ramp (°C/min) | Temperature (°C) | Time (min) |
|---|---|---|---|
| | - | 75 | 2 |
| | 5 | 100 | 7 |
| | 25 | 225 | 1 |

Table S2. GC column retention times for substrates and products

| **Column program** | **Substrate** | $t_R$ **(min)** | **Product** | $t_R$ **(min)** |
|---|---|---|---|---|
| **A** | ketoisophorone | 8.2 | levodione | 12.3 (*R*), 12.7 (*S*) |
| **B** | 2-methyl-*N*-phenylmaleimide | 10.1 | 2-methyl-*N*-phenylsuccinimide | 12.6 |
| **C** | 2-methylcyclohexenone | 14.8 | 2-methylcyclohexanone | 10.9 (*S*), 11.1 (*R*) |

Table S3. GC conversions and enantiomeric excess

| **Substrate/reaction** | **conversion (%)** | ***ee* (%)** |
|---|---|---|
| **ketoisophorone** | | |
| Wild type OYERo2 | 35[b] | 90[a] |
| Mutant OYERo2 | 17[b] | 86[a] |
| **2-methyl-*N*-phenylmaleimide** | | |
| Wild type OYERo2 (NADPH) | 98 | >99 (HPLC) |
| Wild type OYERo2 (BNAH) | 97 | >99 (HPLC) |
| Mutant OYERo2 (NADPH) | 95 | >99 (HPLC) |
| Mutant OYERo2 (BNAH) | 98 | >99 (HPLC) |
| **2-methylcyclohexenone** | | |
| Wild type OYERo2 | 69 | >99 |
| Mutant OYERo2 | 73 | >99 |

[a] Loss of *ee* due to the racemisation of levodione over time.[1] [b] not reliable without a calibration curve, mass balance is off.
